# Supplementary material for: BIN1 gain-of-function in the presynaptic compartment leads to isoform-specific synaptotoxicity
Source: Alzheimers Res Ther. 2026 Jun 3;18:185. doi: 10.1186/s13195-026-02103-7 (PMC13466144; doi:10.1186/s13195-026-02103-7)
Supplement: Supplementary file 1 [file 13195_2026_2103_MOESM1_ESM.pdf]

SUPPLEMENTARY INFORMATION

BIN1 expression in the presynaptic compartment leads to isoform-specific synaptotoxicity

Erwan Lambert[1†], Carla Gelle[1†], Valentin Leclerc[1†], Alejandra Freire-Regatillo[1†], Lucie Liefooghe[1], Nicolas Barois[2,3], Tommy Malfoi[1], Xavier Hermant[1], Florie Demiautte[1], Inès Gallienne[1], Frank Lafont[3], Philippe Amouyel[1], Karine Blary[4], Sabine Kuenen[5,6], Chloé Najdek[1], Patrik Verstreken[5,6], Dolores Siedlecki-Wullich[1], Pierre Yger[7], Jean-Charles Lambert[1], Devrim Kilinc[1]*, Pierre Dourlen[1]*

[1] Université de Lille, Inserm, CHU Lille, Institut Pasteur de Lille, U1167 – RID – AGE – Facteurs de risque et déterminants moléculaires liés au vieillissement, Lille, France

[2] Université de Lille, CNRS, Inserm, CHU Lille, Institut Pasteur de Lille, UAR CNRS 2014 - US Inserm 41 - PLBS, Lille, France.

[3] University of Lille, CNRS, Inserm, CHU Lille, Institut Pasteur Lille, U1019-UMR 9017-CIIL-Center for Infection and Immunity of Lille, Lille, France.

[4] Univ. Lille, CNRS, Centrale Lille, Univ. Polytechnique Hauts-de-France, UMR 8520 - IEMN - Institut d'Electronique de Microélectronique et de Nanotechnologie, F-59000 Lille, France

[5] VIB Center for Neuroscience, Leuven, Belgium

[6] Department of Neurosciences, Leuven Brain Institute, KU Leuven, Leuven, Belgium

[7] Lille Neurosciences & Cognition (lilNCog) – U1172 (INSERM, Lille), Univ Lille, CHU Lille 59045 Lille, France

[†] Equal contribution

* Address for correspondence: devrim.kilinc@pasteur-lille.fr; pierre.dourlen@pasteur-lille.fr

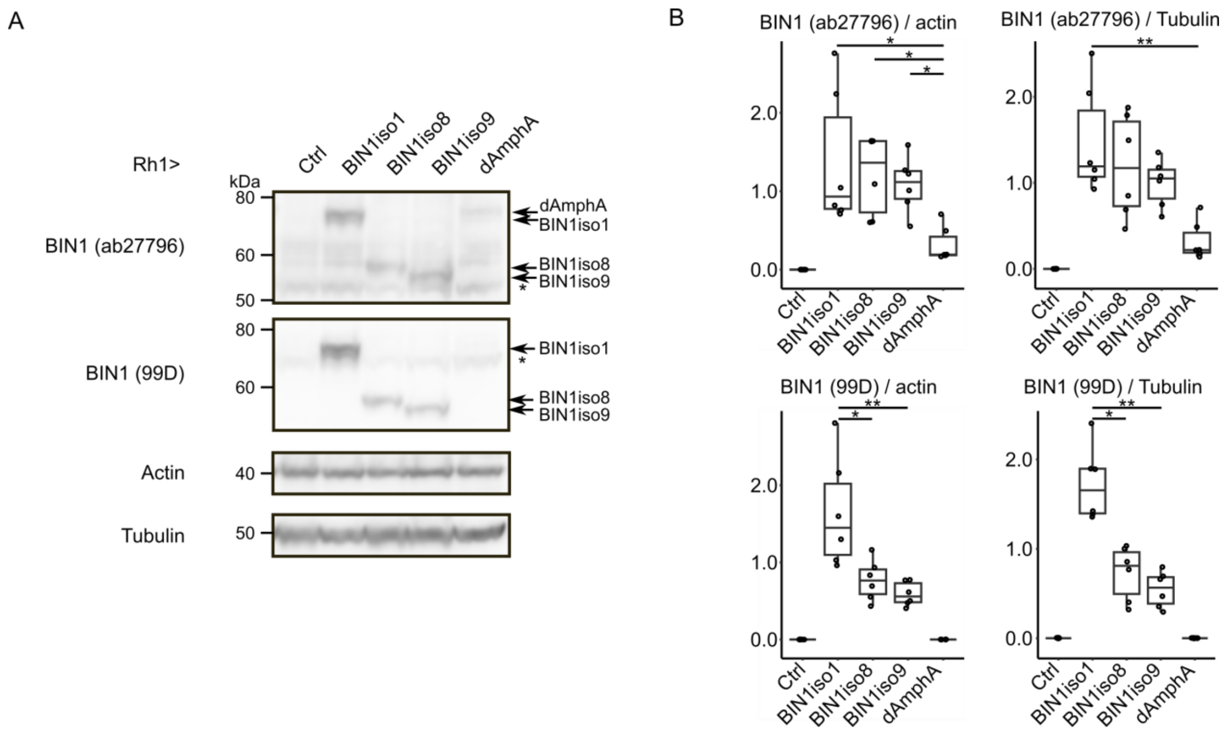

Supplementary Figure 1: Analysis of BIN1 levels in *Drosophila* expressing human BIN1 isoforms and *Drosophila* Amphiphysin isoformA under an Rh1 driver. **A.** Representative western blots analyzing the transgenic expression of human BIN1 isoforms and *Drosophila* Amphiphysin in 1-2-day-old *Drosophila* heads. BIN1 was detected using two antibodies (ab27796 and 99D). Actin and Tubulin were used as loading controls. Non-specific bands were marked with stars. **B.** Quantification of data obtained from 6 independent experiments. Statistical analyses were performed with non-parametric Kruskal Wallis tests followed by Dunn's multiple comparisons (*p*-values adjusted using the Holm method, * $p<0.05$; ** $p<0.01$). The Ctrl group and the Ctrl and dAmphA groups were excluded from the statistical analysis for blots obtained using the BIN1 ab27796 and BIN1 99D antibodies, respectively.

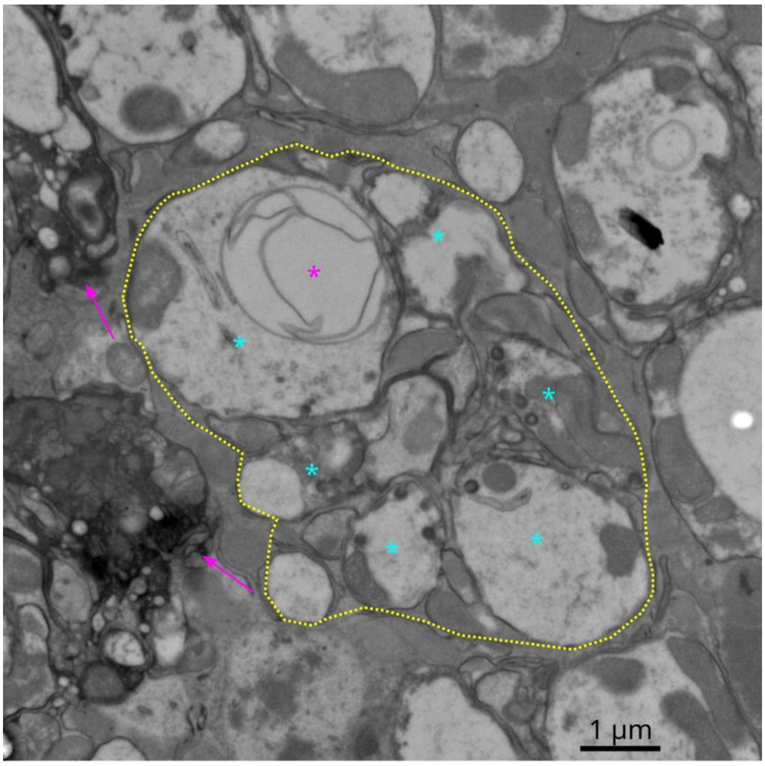

Supplementary Figure 2: Electron microscopy image showing the degeneration of synaptic terminals in *Drosophila* photoreceptor neurons expressing BIN1 iso1 at day 15. Six synaptic terminals (cyan stars) are gathered into a lamina cartridge (yellow dotted line). Expression of BIN1 iso1 resulted in the presence of giant vesicles (magenta star). Magenta arrows point to degenerating synaptic terminals in neighboring cartridges.

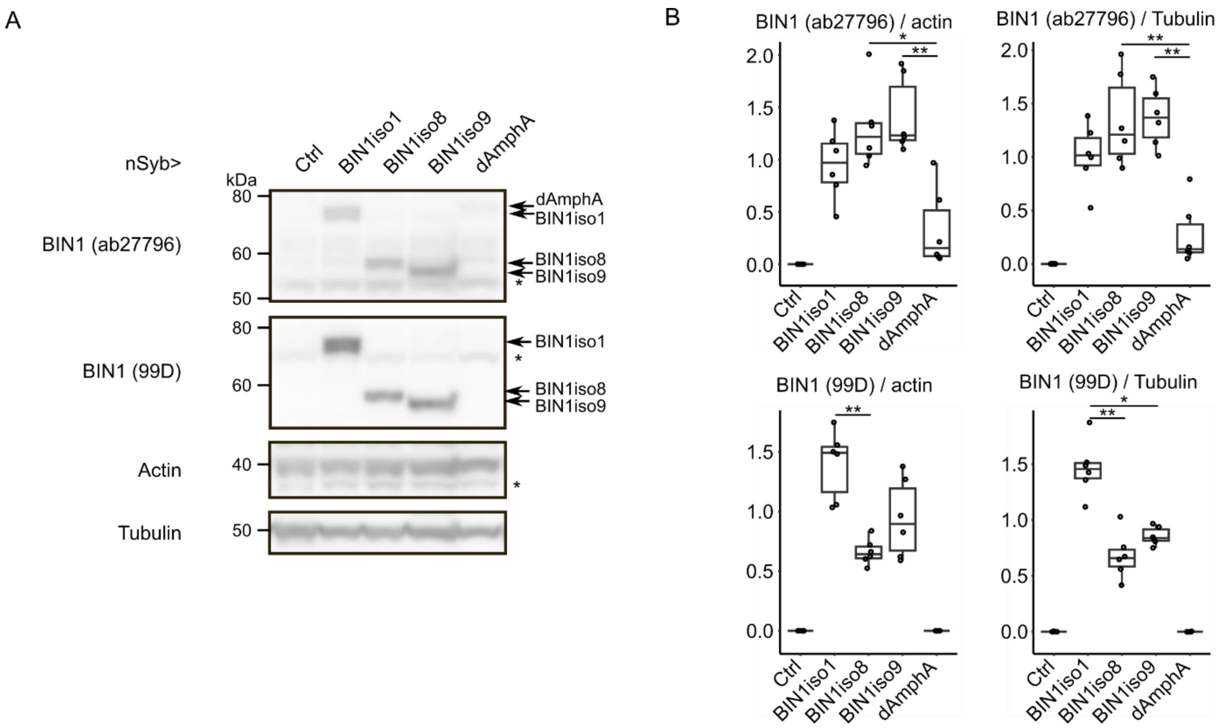

Supplementary Figure 3: Analysis of BIN1 levels in *Drosophila* expressing human BIN1 isoforms and *Drosophila* Amphiphysin under an nSyb driver. **A**. Representative western blots analyzing the transgenic expression of human BIN1 isoforms and *Drosophila* Amphiphysin in 1-2-day-old *Drosophila* heads. BIN1 was detected using two antibodies (ab27796 and 99D). Actin and Tubulin were used as loading controls. Non-specific bands were marked with stars. **B.** Quantification of data obtained from 6 independent experiments. Statistical analyses were performed with non-parametric Kruskal Wallis tests followed by Dunn's multiple comparisons (*p*-values adjusted using the holm method, * $p<0.05$; ** $p<0.01$). The Ctrl group and the Ctrl and dAmphA groups were excluded from the statistical analysis for blots obtained using the BIN1 ab27796 and BIN1 99D antibodies, respectively.

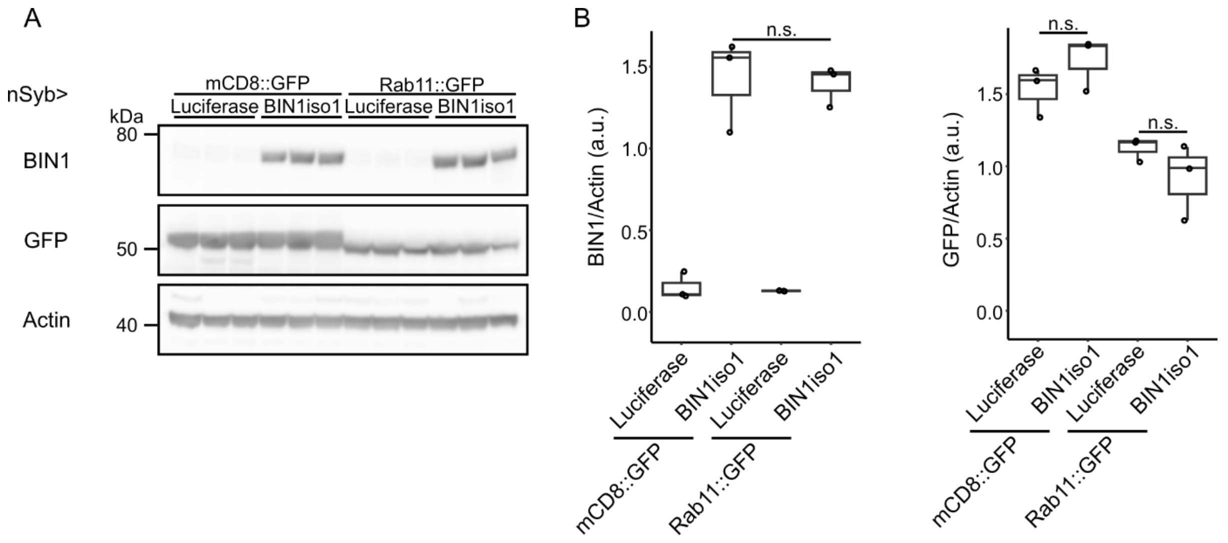

Supplementary Figure 4: Protein levels in *Drosophila* expressing BIN1iso1 and Rab11. **A.** Representative western blots using *Drosophila* head protein extracts from animals expressing luciferase, mCD8::GFP (both used as controls), BIN1iso1 and Rab11::GFP, under an Nsyb driver, using anti-BIN1 (99D), anti-GFP and anti-actin antibodies. **B.** Quantification of BIN1 and GFP from 3 independent experiments. Statistical analyses were performed with non-parametric Kruskal Wallis tests followed by Dunn's multiple comparisons (*p*-values adjusted using the holm method, n.s. not significant).

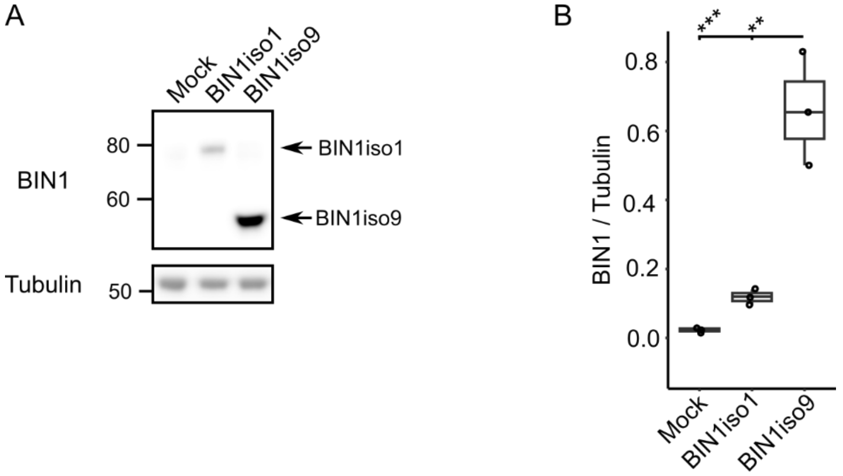

Supplementary Figure 5: Analysis of BIN1 levels in rat primary neuronal culture transduced for human BIN1iso1 and BIN1iso9. **A.** Representative western blots analyzing the transduced expression of human BIN1 isoforms in 21DIV rat primary neuronal culture. BIN1 was detected using 99D antibody (ab27796 and 99D). Tubulin was used as loading control. **B.** Quantification of data obtained from 3 independent experiments. After checking the normality of the data with a Shapiro-Wilk test, the statistical analysis was performed with a parametric ANOVA followed by a Tukey post-hoc multiple comparison (** $p<0.01$; *** $p<0.001$).

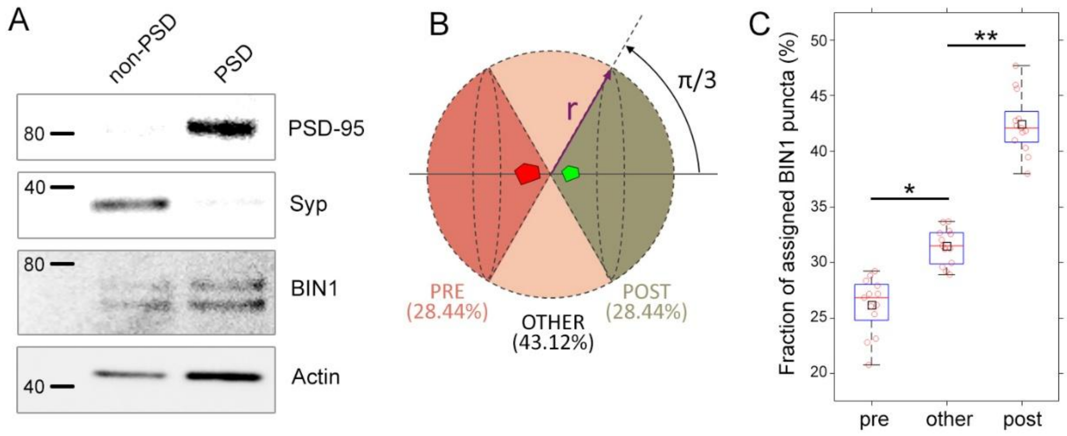

Supplementary Figure 6: Analysis of the localization of BIN1 signals relative to synapses. **A.** Western blot following synaptosomal extraction from rat cortical neuron cultures. The non-PDS fraction is rich in presynaptic marker Synaptophysin1 (Syp) and the PSD fraction is rich in postsynaptic marker PSD-95 as well as actin, as expected. BIN1 is detected in both fractions, with a higher abundance in the PSD fraction. **B.** Visual representation of the relative localization analysis. The center of the sphere refers to the center of the synapse, as defined by the middle point between the presynaptic marker (red) and the postsynaptic marker (green) that are positioned within 1 µm from each other. The cut-off distance ***r*** for assigning a BIN1 spot to a synapse was 1.5 µm in this study. Pre- and postsynaptic zones (indicated as brown and olive shading, respectively) were defined as cones with apexes at the center of the sphere and with cone angle of 60° (π/3) plus the spherical caps. The corresponding volume fractions of the pre- and postsynaptic zones and the remaining volume of the sphere (other) are given. **C.** Fraction of BIN1 puncta assigned to synapses in these three zones. In box plots, red bars and black squares indicate sample median and mean, respectively. Each data point represents one microfluidic device. N=13 devices from three independent cultures. Kruskal–Wallis test with Tukey-Kramer correction. * $p<0.05$; ** $p<0.01$.

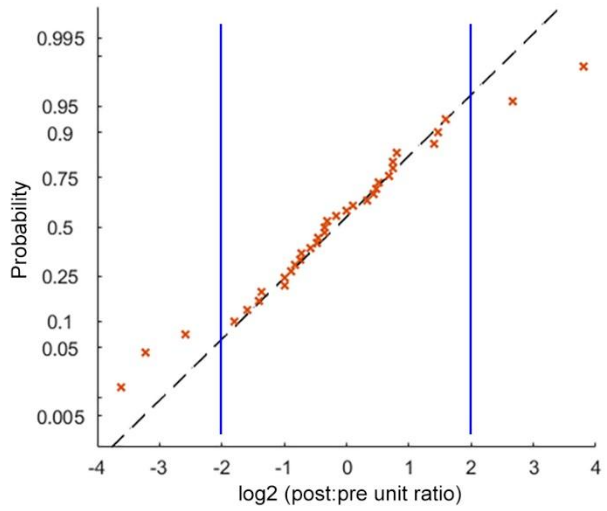

Supplementary Figure 7: Probability plot comparing the distribution of the fold differences between unit numbers detected in presynaptic and postsynaptic chambers to the normal distribution. Each point indicates one MEA-integrated microfluidic device, in which the spike sorting algorithm detected at least one unit in both chambers (n = 35 devices from 7 independent cultures). Dashed line passes through the lower and upper quartiles. Solid lines indicate the limits for 4-fold difference in unit numbers and correspond to the exclusion criteria used for this dataset (|*z*-score| = 1.5).

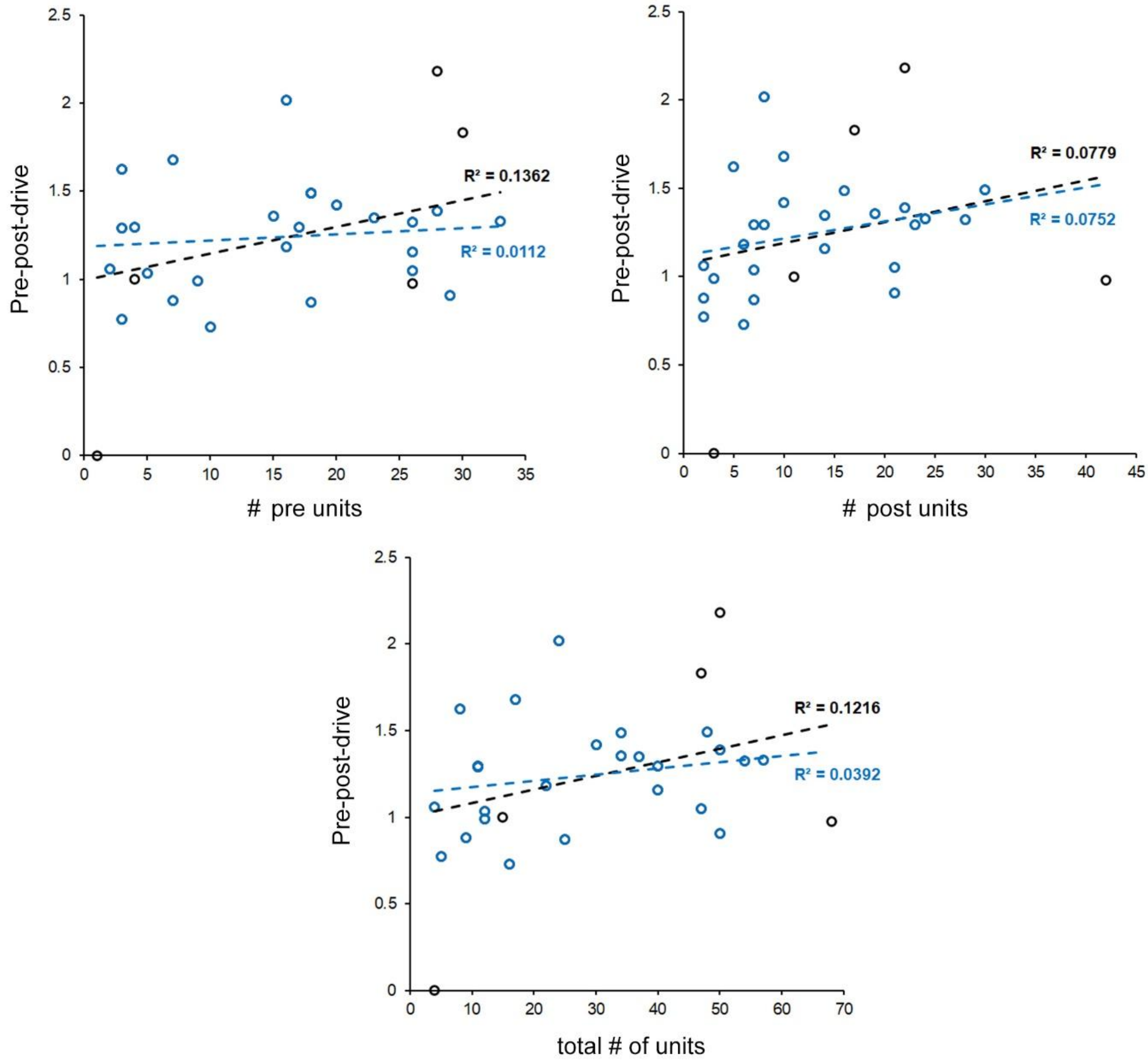

Supplementary Figure 8: Correlation between detected unit numbers and *pre-post-drive*. Each point indicates one MEA-integrated microfluidic device within the normal distribution of fold differences in unit numbers (n = 30 devices from 7 independent cultures). Outlier data points (black dots) were included for clarity. Dashed lines show the least-squares fits before (black) and after (blue) outlier exclusion.
